# Economy of Strength

**Published:** 1874-11

**Authors:** 


### ECONOMY OF STRENGTH.

Most persons can do more work, with less fatigue, any day by being a little thoughtful and more observant of the instincts. Look at any dozen men crossing a street leading out of Broadway while a vehicle is passing before them. Very many will, instead of stopping still until the carriage has passed, slow their pace, and make a circuit around the end of the vehicle. This requires more steps, but instinct teaches that less exertive power is demanded to keep moving, than by coming to a dead standstill, to set the body in motion again. It is the starting after frequent stoppings which kill our stage and car horses in four years. With only one start in four they would travel farther, and live a year or two longer. Hence, every well-meaning man, in mercy to the horse, should only slow a car when wishing to get off; and ladies, always tender-hearted, should be careful to rather walk a few yards than to stop a car between two blocks. Last winter a Fourth Avenue car, between two crossings at the Methodist Church, was brought to a dead stop four times, with forty-eight passengers twice counted.

In mid-winter the news boys scream out at the top of their voices: "Here's the Herald, Times, Tribune, and World;" in July it is condensed to "Morning Papers." This is instinct, adapting the requisite expenditure of strength to the circumstances of the season. None of us walk as fast in Summer as in Winter.

When the first cholera was present in New York, the carpenters were noticed, in nailing down floors, to strike but one stroke of the hammer, and rest with the face of the hammer on the floor, instead of three strokes without a pause, as is the case in freezing, cold weather. The old man instinctively lets down the bars, or goes a long way round, instead of springing over at a bound.

We get up in the morning with a certain amount of strength, and much may be gained by economizing, in every way possible, during the day. Very many persons, for want of thought, squander a great deal of vital power. The cook should do all she can sitting down, as less strength is expended in that position than in standing. She should peel her vegetables, string her beans, wash and wipe her dirty hands in a sitting position.

As long as thirty years ago, it was an interesting sight to see letter-carriers of the London Post Office leave the building with their letters in a long carriage, the man who was to get out first, sitting next the door.

Mr. Sullivan, of Illinois, one of the most extensive and successful farmers of the State, required his men to ride to their fields of labor. The clergyman should neither sing nor speak, nor converse above the most ordinary tones until he enters the pulpit, and even then he should save his voice for the sermon, being careful, however, to remember not to make an effort to speak loud, but rather to enunciate distinctly, cutting off each word clear and sharp before beginning another. Many a good sermon is lost to persons farthest from the speaker for want of attention to the fact in acoustics, that the shrill, small voice is often heard with greater distinctness than the muttering thunder of a careless speaker.

